# Hydroethanolic Extract of *Fritillariae thunbergii* Bulbus Alleviates Dextran Sulfate Sodium-Induced Ulcerative Colitis by Enhancing Intestinal Barrier Integrity

**DOI:** 10.3390/nu15122810

**Published:** 2023-06-20

**Authors:** Ami Lee, You Chul Chung, Kwang-Youn Kim, Chan Ho Jang, Kwang Hoon Song, Youn-Hwan Hwang

**Affiliations:** 1 Herbal Medicine Research Division, Korea Institution of Oriental Medicine, Daejeon 34054, Republic of Korea; 2 Korean Convergence Medical Science Major, KIOM School, University of Science & Technology (UST), Daejeon 34054, Republic of Korea; 3 Korean Medicine (KM)-Application Center, Korea Institute of Oriental Medicine (KIOM), Daegu 41062, Republic of Korea

**Keywords:** *Fritillariae thunbergii* Bulbus, ulcerative colitis, gastrointestinal tract, tight junction

## Abstract

The incidence of ulcerative colitis (UC), an inflammatory disorder of the gastrointestinal tract, has rapidly increased in Asian countries over several decades. To overcome the limitations of conventional drug therapies, including biologics for UC management, the development of herbal medicine-derived products has received continuous attention. In this study, we evaluated the beneficial effects of a hydroethanolic extract of *Fritillariae thunbergii* Bulbus (FTB) in a mouse model of DSS-induced UC. The DSS treatment successfully induced severe colonic inflammation and ulceration. However, the severity of colitis was reduced by the oral administration of FTB. Histopathological examination showed that FTB alleviated the infiltration of inflammatory cells (e.g., neutrophils and macrophages), damage to epithelial and goblet cells in the colonic mucosal layer, and fibrotic lesions. Additionally, FTB markedly reduced the gene expression of proinflammatory cytokines and extracellular matrix remodeling. Immunohistochemical analysis showed that FTB alleviated the decrease in occludin and zonula occludens-1 expression induced by DSS. In a Caco-2 monolayer system, FTB treatment improved intestinal barrier permeability in a dose-dependent manner and increased tight junction expression. Overall, FTB has potential as a therapeutic agent through the improvement of tissue damage and inflammation severity through the modulation of intestinal barrier integrity.

## 1. Introduction

Inflammatory bowel disease (IBD), comprising ulcerative colitis (UC) and Crohn’s disease, is a chronic remission or progressive inflammation affecting the entire gastrointestinal tract and colonic mucosa [1]. While morbidity rates have stabilized in Western countries, newly industrialized countries have experienced rapid increases in the incidence rates of IBD since the beginning of the 21st century [2]. Symptoms of IBD include chronic diarrhea, abdominal pain, rectal bleeding, weight loss, and colonic shortening [3]. UC is the result of a complex interplay of various factors, such as environmental factors, genetic factors, oxidative damage, changes in the gut microbiome, and immune system disorders. Corticosteroids, immunomodulators, 5-aminosalicylic acid (mesalamine), and biological agents (e.g., adalimumab) are currently used for UC treatment. These drugs provide clinical relief from UC, but after a period they lose their effectiveness and can cause multiple side effects. The drugs primarily focus on lowering the level of inflammation [4]. Conversely, enhancing barrier function can inhibit UC by inhibiting excessive compensatory immune responses due to multiple levels of protective mechanisms [5,6]. The intestinal barrier helps to maintain mucosal homeostasis by bridging the gap between the gut microbiome and the gut immune system. A damaged intestinal barrier is often associated with various gastrointestinal disorders, including UC. The downregulation of epithelial cell tight junction (TJ) protein expression in the intestinal barrier can increase intestinal permeability, allowing harmful substances (e.g., pathogens and endotoxins) to pass through the intestinal mucosa into the blood [7]. Shah et al. reported that patients with UC who achieved mucosal healing demonstrated long-term clinical remission compared to those without mucosal healing [8]. Thus, maintaining proper intestinal barrier function may be a crucial goal in UC treatment. However, currently available therapies do not specifically modulate epithelial barrier function [9]; hence, there is a pressing need to develop more effective and promising therapies for UC.

The genus Fritillariae is one of the largest genera in the Liliaceae family, with a history of use as a source of pharmacological properties for thousands of years [10]. *F. thunbergii*, a plant native to many Asian countries such as Korea, China, and Japan [11], is cultivated in these regions and European countries as an ornamental plant [12]. *F. thunbergii* Bulbus, also known as “Jeol-Pae-Mo” in Korea, is the bulb of *F. thunbergii*, widely used as medicine and food globally [13,14,15]. The roasted bulbs of Fritillaria species were used as food by Native Americans [15]. In traditional oriental medicine, *F. thunbergii* Bulbus has protective properties for the lungs and heart due to its cold properties and bitter taste [10], and it is primarily used as an expectorant and antitussive. It has also been used to treat skin diseases such as skin ulcers and dermatitis [14,16].

Several phytochemical components of *F. thunbergii* Bulbus, including alkaloids such as sipeimine, peiminine, and yibeissine, have been identified through phytochemical studies [13,14,17]. *F. thunbergii* Bulbus reportedly possess pharmacological properties, including anticancer, bronchial relaxation, antitussive, and anti-inflammatory effects. The efficacy of anti-inflammatory and pain suppression effects of phytochemicals has been studied, with research receiving increased attention [10,18,19,20]. Although multiple pharmacological efficacies have been reported in the various fields described above, the pharmacological efficacy for IBD, especially UC, has not been studied.

In this study, we examined the beneficial effects of a hydroethanolic extract of *F. thunbergii* bulbus (FTB) against DSS-induced experimental ulcerative colitis. We found that FTB effectively reduced tissue damage in the colon and inflammation by enhancing barrier integrity in a DSS-induced ulcerative colitis mouse model. Furthermore, FTB increased TJ expression and effectively lowered monolayer permeability in vitro. Here, we report for the first time the efficacy of FTB against induced UC.

## 2. Materials and Methods

### 2.1. Chemicals and Reagents

All reference standards, except for xanthurenic acid (sipeimine, peimisine, peimine, peiminine, and solanidine), were purchased from ChemFaces (Wuhan, China), while xanthurenic acid was obtained from Targetmol (Wellesley Hills, MA, USA). LC-MS-grade mobile phases (water, acetonitrile, and formic acid) were procured from Fisher Chemical (Waltham, MA, USA) for UPLC-tandem mass spectroscopy. HyClone (Logan, UT, USA) supplied the cell culture medium, fetal bovine serum (FBS), and penicillin/streptomycin solution, and Sigma-Aldrich (St. Louis, MO, USA) provided the FITC-conjugated dextran probe (FD-4). All antibodies used in Western blot analysis were obtained from Cell Signaling Technology (Danvers, MA, USA).

### 2.2. Preparation of FTB

The hydroethanolic extract of *F. thunbergii* bulbus was procured from the National Development Institute of Korean Medicine (Gyeongsan, Republic of Korea). *F. thunbergii* Bulbus (500 g) was prepared from FTB via reflux extraction with 70% ethanol (3.5 L) in water for 3 h. The extract was filtered via qualitative filter paper, grade 2 (Whatman Limited, Maidstone, UK), and dried using a vacuum freeze-dryer (Ilshin Biobase, Gyeonggi-do, Republic of Korea); the lyophilized powder of FBT was stored at −20 °C before use.

### 2.3. Identification of Phytochemicals Using Ultra-High Performance Liquid Chromatography-Tandem Mass Spectroscopy (UPLC-MS/MS)

To analyze the FTB, a Dionex UltiMate 3000 UPLC system equipped with a Thermo Q-Exactive mass spectrometer was used. Chromatographic separation was performed using an Acquity BEH C18 column (100 × 2.1 mm, 1.7 µm) and the gradient setting was performed as described by Shim et al., using 0.1% formic acid in water and acetonitrile [21]. The identification of phytochemicals in FTB was performed by comparing their retention time (Rt) and mass spectral data with reference standards or based on a previous report [21,22]. Further details can be found in the Supplementary Materials.

### 2.4. Animal Study

#### 2.4.1. Animals

This study was approved by the Institutional Animal Care and Use Committee of the Korea Institute of Oriental Medicine (permit number: 21-110, Daejeon, Republic of Korea). Six-week-old male C57BL/6 mice (20 to 23.5 g body weight) were purchased from DooYeol Biotech (Seoul, Republic of Korea), housed under standard laboratory conditions with free access to water and food, and acclimated for seven days.

#### 2.4.2. Animal Group and Administrations

The mice were randomly allocated into five groups (N = 5/group): normal, negative control (N.C.), positive control (P.C.), low-dose FTB (FTB-L), and high-dose FTB (FTB-H). The normal group was provided with drinking water, while the other groups received 4% (*w*/*v*) dextran sodium sulfate (DSS; MW 36–50 kDa; MP Biomedicals, Solon, OH, USA) added to their drinking water for 12 days to induce experimental colitis. Mice in the normal and N.C. groups were orally administered distilled water; those in the P.C. group received mesalamine (200 mg/kg BW) suspended in distilled water; and those in the FTB-L and FTB-H groups were administered FTB (100 and 200 mg/kg BW, respectively) dissolved in distilled water during DSS treatment. Following treatment, the mice were euthanized simultaneously using avertin (Sigma-Aldrich), and the blood, spleen, and colon were collected, with serum separated using a BD Microtainer (BD, San Jose, CA, USA).

#### 2.4.3. Colitis Severity Evaluation and Histopathological Analysis

The disease activity index (DAI) was assessed daily after DSS treatment. Details of the evaluation criteria are described in the Supplementary Materials. Following the 12-day treatment period, the spleen weight and colon length from the appendix to the anus were measured. For histopathological evaluation, the colonic segments were fixed in 10% neutral-buffered formalin, and the paraffin-embedded colon samples were sectioned and stained with hematoxylin and eosin (H&E), Masson’s trichrome (MT), and Periodic Acid Schiff (PAS). The stained images were scanned using a MoticEasyScan digital slide scanner (Motic, Richmond, BC, Canada). The histopathological severity was graded according to a scoring system described by Cooper and Dieleman [23,24]. The scoring system was assessed with scores ranging from 0 to 3 for the amount and extent of inflammation and scores ranging from 0 to 4 for crypt damage. The total histopathological score was calculated as the sum of each feature score (severity of inflammation, extent of inflammation, and crypt damage) multiplied by the score of percentage of involvement. In the MT-stained image, the ratio of the Masson-positive area (i.e., the area stained blue by Masson’s trichrome) in the entire colon tissue area of each sample was measured and converted into a fold value compared to the normal group. In the PAS-stained images, the number of goblet cells per 10 crypt units was counted. Image analysis and editing were performed using the Motic digital slide assistant (Motic) and Fiji [25].

#### 2.4.4. Immunohistochemistry (IHC)

Antigen retrieval of Myeloperoxidase (MPO) and ZO-1 was achieved by boiling in citrate buffer (pH 6.0) and antigen retrieval of F4/80 and occludin was performed by boiling them in Tris-EDTA buffer (pH 9.0). After blocking, all sections were incubated with primary antibodies against MPO (1:400 dilution, Abnova, PAB7992, Taipei, Taiwan), F4/80 (1:600 dilution, Cell Signalling, cs-70076, Danvers, MA, USA), ZO-1 (1:250 dilution, Abcam, ab96587, San Francisco, CA, USA), and occludin (1:200 dilution, Abcam, ab216327) at appropriate temperatures and times. An immune response was then induced using the ImmPRESS^®^ HRP Goat Anti-Rabbit IgG Polymer Detection Kit, peroxidase (Vector Laboratories, MP-7451, Burlingame, CA, USA). The DAB Chromogen/Substrate Kit (High Contrast) (Scytek, UT, USA) was used for visualization, and counterstaining was performed with hematoxylin. Image scanning, analysis, and editing were performed as described in Section 2.4.3.

#### 2.4.5. Real-Time PCR

Full details are described in the Supplementary Materials.

### 2.5. In Vitro Study

#### 2.5.1. Cell Culture and Viability

The Caco-2 cells from the American Type Culture Collection (Manassas, VA, USA) were cultured in Eagle’s minimum essential medium supplemented with 10% FBS, 100 U/mL penicillin, and 100 μg/mL streptomycin at 37 °C in an atmosphere of 5% CO_2_. Cell viability was evaluated using Cell Counting Kit-8 (CCK-8) (Dojindo Molecular Technologies Inc., Rockville, MD, USA), as described by Kim et al. [26]. Cell viability was calculated relative to untreated controls. Results were obtained from three independent experiments.

#### 2.5.2. Measurement of Transepithelial Electrical Resistance (TEER) and Epithelial Paracellular Permeability

Caco-2 cells were seeded at 1 × 10^5^ cells/insert in an area of 0.33 cm^2^ polyethylene terephthalate membrane with 0.4 μm pores (Millipore, Bedford, MA, USA). The medium was changed every two days for a total of 21 days to allow for complete differentiation. The cells were pretreated with multiple doses of FTB (0, 6.25, 12.5, and 25 μg/mL) for 1 h before being exposed to 50 ng/mL IL-6. The electrical resistance was measured in triplicate using a Millicell ERS-2 Voltohmmeter (Millipore) at 24 h and was expressed as Ω·cm^2^. Paracellular permeability was assessed using FD-4. After pretreatment, the apical and basolateral sections were washed with PBS, followed by adding 1 mg/mL of FD-4 to the apical side and PBS to the basolateral side. The cells were incubated at 37 °C for 1 h, and 100 μL of the basolateral side medium was measured at excitation and emission wavelengths of 490 and 520 nm using a VersaMax microplate reader (Molecular Devices LLC, Sunnyvale, CA, USA). Results were obtained from three independent experiments.

#### 2.5.3. Western Blot Analysis

Western blot analysis was used to evaluate TJ protein expression in a Caco-2 monolayer system. Full details are described in the Supplementary Materials.

### 2.6. Statistical Analysis

The data are presented as the mean ± standard error. Statistical analyses were performed using GraphPad Prism 9 (GraphPad Software, San Diego, CA, USA). One-way ANOVA with Dunnett’s post-hoc test and Student’s *t*-test were performed to determine statistical significance, represented by the *p*-values: * *p* < 0.05, ** *p* < 0.01, and *** *p* < 0.001.

## 3. Results

### 3.1. Nine Phytochemicals of FTB Were Identified

Ultra-high-performance liquid chromatography-tandem mass spectroscopy (UPLC-MS/MS) is a highly precise analytical technique that provides extensive chemical profiling. It is considered to be one of the most accurate and important assays for active ingredient characterization in herbal medicine [27]. In this study, UPLC-tandem mass spectrometry analysis was utilized to characterize the components of FTB. Using retention time (Rt) and mass spectral data from reference standards and reported studies, eight alkaloids (yibeissine, peiminoside, sipeimine glucoside, sipeimine, peimisine, peimine, peiminine, and solanidine) and xanthurenic acid, which are basic components of FTB, were identified in FTB (Table 1 and Supplementary Figure S1) [21,22].

### 3.2. FTB Improves Clinical Symptoms of DSS-Induced Ulcerative Colitis

**Figure 1:**
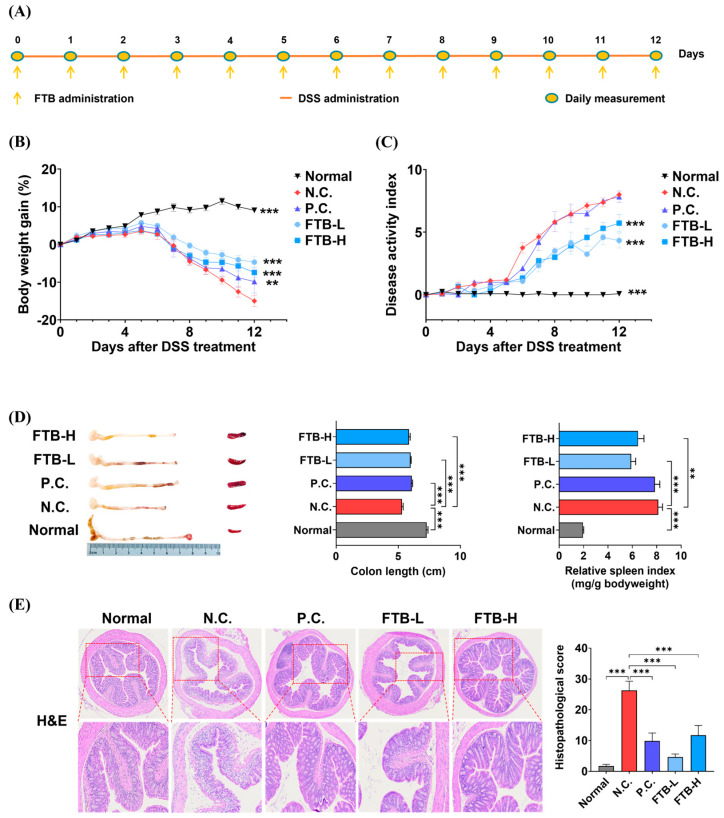
Therapeutic effects of FTB on dextran sulfate sodium (DSS)-induced colitis: (**A**) Scheme of the animal experiment protocol in the present study; (**B**) Measured body weight during 4% DSS treatment for 12 days (N = 7–8); (**C**) Calculated disease activity index (DAI) value described in Material and Methods; (**D**) Comparison of colon length and spleen size (N = 7–8); (**E**) Colon tissue after H&E staining and histopathological score calculated using the described method in Material and Methods (N = 5). All data were analyzed using one-way ANOVA with Dunnett’s post-hoc test compared to N.C. Data are shown as mean ± SEM value. N.C., negative control group; P.C., positive control group; FTB, hydroethanolic extract of *F. thunbergii* Bulbus; FTB-L, low dose (100 mg/kg BW) of FTB; FTB-H, high dose (200 mg/kg BW) of FTB. ** *p* < 0.01, *** *p* < 0.001.

To assess the therapeutic efficacy of FTB in colitis, we induced colitis in mice using 4% DSS for 12 days, while simultaneously administering FTB orally. The N.C. group demonstrated a continuous loss of body weight, accompanied by severe diarrhea and fecal occult blood, in comparison to the normal group (*p* < 0.001). However, mice treated with mesalamine and FTB showed a reduction in body weight loss when compared to the N.C. group (*p* < 0.001). There was no significant difference in the DAI value between the P.C. group and the N.C. group (Figure 1B,C). Moreover, DSS administration can lead to colon shortening and spleen enlargement. The N.C. group exhibited a shorter colon length and a larger spleen size than the normal group (*p* < 0.001). In contrast, the P.C., FTB-L, and FTB-H groups demonstrated significantly longer colons (*p* < 0.001). Additionally, the FTB-administered group showed a smaller spleen size than the N.C. group (FTB-L, *p* < 0.001; FTB-H, *p* < 0.01; Figure 1D). The colon tissue of mice in the N.C. group was severely damaged, and the pathological changes were quite evident. The histopathological score of the N.C. group was approximately 15 times higher than that of the normal group (*p* < 0.001), while mice treated with mesalamine and FTB showed lower histopathological scores than the N.C. group (*p* < 0.001, Figure 1E).

### 3.3. FTB Improves Clinical Symptoms of DSS-Induced Ulcerative Colitis and Reduces Inflammation in the Colon Tissue of DSS-Induced Ulcerative Colitis

**Figure 2:**
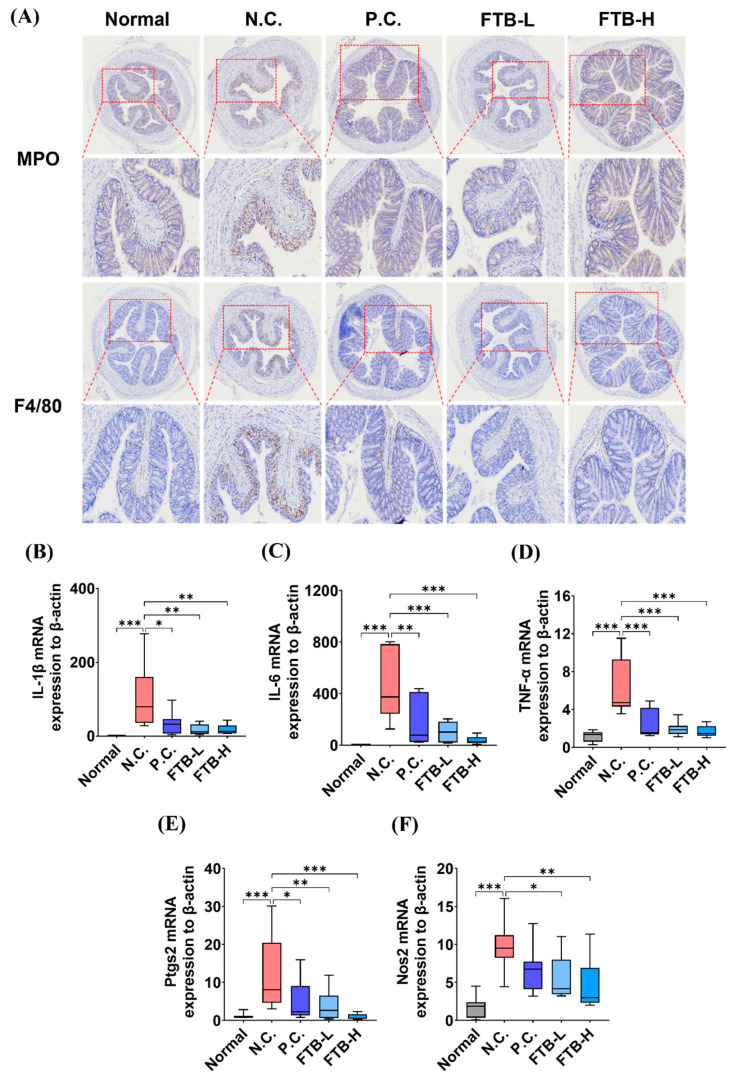
Effects of FTB on the distribution of inflammatory markers in the colons of DSS-induced ulcerative colitis mice: (**A**) IHC staining-related immune cells in colon tissue of DSS-induced ulcerative colitis mice (N = 5); (**B**–**F**) The mRNA expression of interleukin (IL)-1β, IL-6, TNF-α, COX-2 (Ptgs2), and iNOS (NOS2) in colon tissue of DSS-induced ulcerative colitis mice analyzed using real-time PCR (N = 7–8). All data were analyzed using one-way ANOVA with Dunnett’s post-hoc test compared to N.C. Data are shown as mean ± SEM value. N.C., negative control group; P.C., positive control group; FTB, hydroethanolic extract of *F. thunbergii* Bulbus; FTB-L, low dose (100 mg/kg BW) of FTB; FTB-H, high dose (200 mg/kg BW) of FTB. * *p* < 0.05, ** *p* < 0.01, *** *p* < 0.001.

Neutrophils and macrophages were immunostained using the markers myeloperoxidase (MPO) and F4/80, respectively, to examine the impact of FTB on inflammation-related cell accumulation in the colon of a DSS-induced ulcerative colitis mouse model. The N.C. group displayed a noticeable accumulation of inflammatory cells compared to the normal group, which was restored by FTB (Figure 2A). Real-time PCR was performed to compare the mRNA expression levels of inflammation-related factors in the colon. In the colon tissue, the mRNA expression of proinflammatory cytokines such as IL-1β, IL-6, and TNF-α was significantly higher in the N.C. group than in the normal group (*p* < 0.001). In contrast, the mRNA expression decreased in the P.C. group (IL-1β, *p* < 0.05; IL-6, *p* < 0.01; TNF-α, *p* < 0.001). The FTB-treated group also showed a significant reduction in the mRNA expression of proinflammatory cytokines (IL-1β, *p* < 0.01; IL-6 and TNF-α, *p* < 0.001; Figure 2B–D). Additionally, the mRNA expression levels of COX-2 and iNOS, markers of inflammation, were compared. Similarly, the mRNA expression of COX-2 and iNOS in the colon tissue was significantly higher in the N.C. group than the normal group (*p* < 0.001), and the expression of COX-2 mRNA decreased in the P.C. group (*p* < 0.05). The FTB-treated groups also showed significantly reduced mRNA expression of COX-2 (FTB-L, *p* < 0.01; FTB-H, *p* < 0.001; Figure 2E). Treatment with FTB resulted in a significant reduction in iNOS mRNA expression in the FTB-L (*p* < 0.05) and FTB-H (*p* < 0.01) groups (Figure 2F).

### 3.4. FTB Prevents the Loss of Tight Junction Protein in the Colon of DSS-Induced Ulcerative Colitis Mice

**Figure 3:**
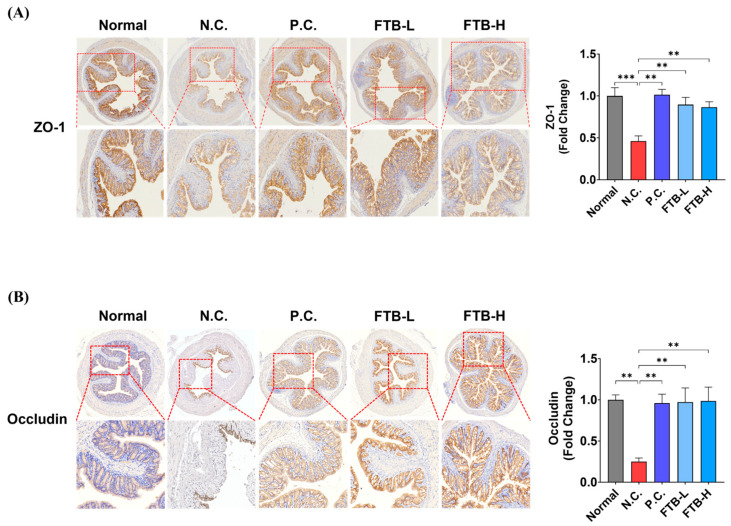
Expression of tight junction proteins in the colons of DSS-induced colitis mice (N = 5): (**A**) IHC staining showing the expression of ZO-1; (**B**) IHC staining showing the expression of occludin. All data were analyzed using one-way ANOVA with Dunnett’s post-hoc test compared to N.C. Data are shown as mean ± SEM value. N.C., negative control group; P.C., positive control group; FTB, hydroethanolic extract of *F. thunbergii* Bulbus; FTB-L, low dose (100 mg/kg BW) of FTB; FTB-H, high dose (200 mg/kg BW) of FTB. ** *p* < 0.01, *** *p* < 0.001.

An investigation of the distribution of TJ proteins in the colon tissue of DSS-induced ulcerative colitis mice was carried out using IHC of ZO-1 and occludin. The results indicated a significant reduction in ZO-1 and occludin levels in the N.C. group compared to the normal group in colon tissue (ZO-1, *p* < 0.001; occludin, *p* < 0.05; Figure 3). However, in the P.C. group, mesalamine, ZO-1, and occludin levels were found to have increased compared to those in the N.C. group (*p* < 0.01). Furthermore, in the FTB-administered group, the expression of ZO-1 and occludin was observed to be higher than that in the N.C. group (*p* < 0.01).

### 3.5. FTB Inhibits Fibrosis by Delaying Extracellular Matrix (ECM) Remodeling and the Collapse of Intestinal Mucosal Layers in the Colon Tissue of DSS-Induced Ulcerative Colitis

**Figure 4:**
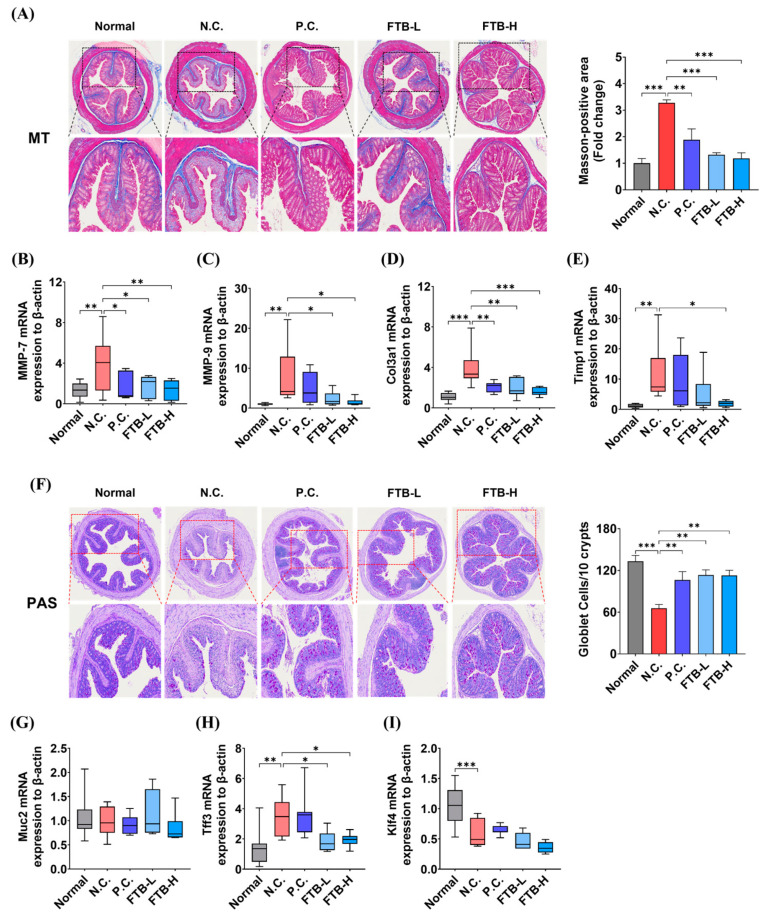
Effects of FTB on extracellular matrix (ECM) remodeling and intestinal mucosa in the colons of DSS-induced ulcerative colitis mice: (**A**) Masson’s trichrome (MT)-stained histological images of colon tissue and measured Masson-positive area (N = 5); (**B**–**E**) The mRNA expression related to ECM remodeling, matrix metallopeptidase (MMP)-7 (Mmp7), MMP9 (Mmp-9), collagen type III alpha 1 (Col3a1), and tissue inhibitor of metalloproteinase 1 (Timp1) (N = 7–8); (**F**) Periodic Acid Schiff (PAS)-stained histological images of colon tissue and counted goblet cells per 10 crypt unit (N = 5); (**G**–**I**) The mRNA expression of mucin 2 (MUC2), trefoil factor 3 (Tff3), and Kruppel-like factor 4 (Klf4) in colon tissue of DSS-induced ulcerative colitis mice was analyzed using real-time PCR (N = 7–8). All data were analyzed using one-way ANOVA with Dunnett’s post-hoc test compared to N.C. The real-time PCR results represent the mean ± SEM values of three independent experiments. N.C., negative control group; P.C., positive control group; FTB, hydroethanolic extract of *F. thunbergii* Bulbus; FTB-L, low dose (100 mg/kg BW) of FTB; FTB-H, high dose (200 mg/kg BW) of FTB. * *p* < 0.05, ** *p* < 0.01, *** *p* < 0.001.

We assessed the histopathological alterations in ulcerative colitis induced by DSS and found that the severity of fibrosis and loss of goblet cells were more pronounced in the N.C. group than in the normal group. Notably, the degree of fibrosis was significantly decreased in the FTB-administered groups compared to that in the N.C. group (*p* < 0.001, Figure 4A). Furthermore, we conducted real-time PCR to evaluate the mRNA expression of ECM remodeling-related factors in colon tissue. The expression of MMP-7, MMP-9, and Timp-1 genes in the N.C. group was higher than in the normal group (*p* < 0.01). Additionally, collagen type III alpha 1 (Col3a1) also had significantly higher mRNA expression in the N.C. group (*p* < 0.001). However, FTB significantly lowered their mRNA expression levels. Moreover, MMP-7, MMP-9, and Col3a1 had lower mRNA expression in a concentration-dependent manner in the FTB-L and FTB-H groups, while TIMP-1 showed low levels only in the FTB-H group (Figure 4B–E).

Additionally, we assessed the status of the intestinal mucous layer by quantifying mucin-containing goblet cells in PAS-stained colon tissue. We found a significant decrease in mucin-containing goblet cells per 10 crypt units in the N.C. group compared to the normal group (*p* < 0.001), which was recovered in the FTB-treated groups (*p* < 0.01, Figure 4F). Furthermore, the mRNA expression of intestinal mucosal-related factors did not differ significantly between all groups for Muc-2 (Figure 4G). The mRNA expression of trefoil factor 3 (Tff3) was higher in the N.C. group than in the normal group (*p* < 0.01), while Kruppel-like factor 4 (Klf4) was decreased (*p* < 0.001). However, FTB suppressed the mRNA expression of Tff3 compared to the N.C. group (*p* < 0.05, Figure 4H), and Klf4 mRNA expression did not seem to be affected by FTB (Figure 4I).

### 3.6. Impact of FTB on Intestinal Barrier Function in a Barrier Model In Vitro

**Figure 5:**
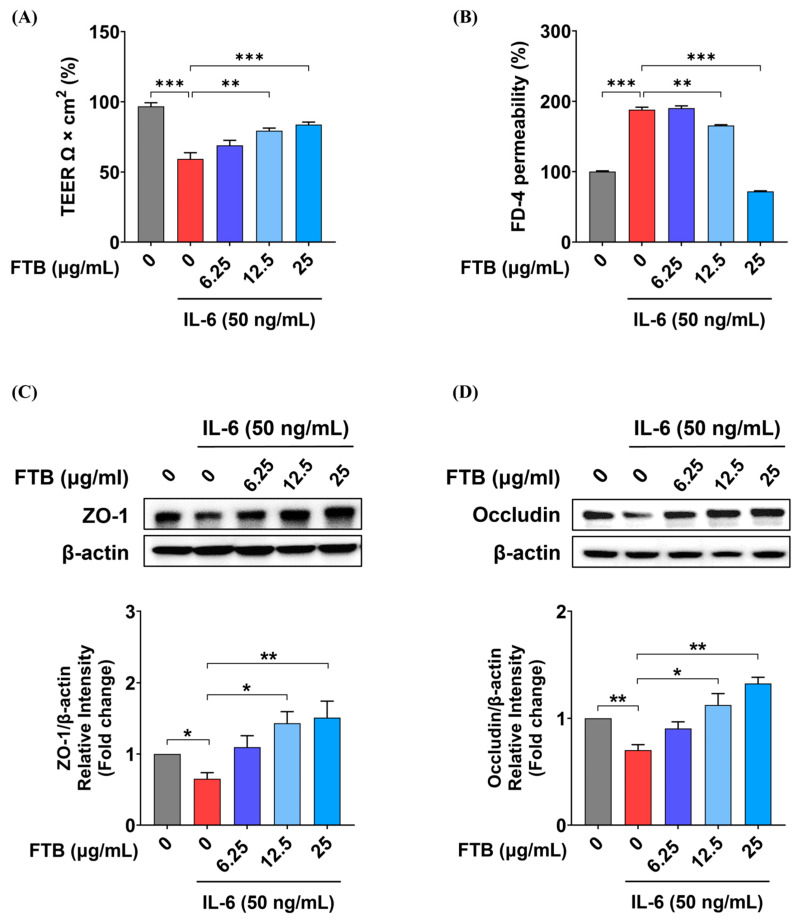
Effect of FTB on Caco-2 monolayer intestinal function: (**A**) Transepithelial Electrical Resistance (TEER); (**B**) Epithelial Paracellular Permeability; (**C**) Representative expressions determined using Western blot analysis for ZO-1 and densitometry of ZO-1 protein expression. β-actin was used as the protein loading control; (**D**) Representative expressions determined using Western blot analysis for occludin and densitometry of occludin protein expression proteins. β-actin was used as the protein loading control; Data were analyzed using one-way ANOVA with Dunnett’s post-hoc test and student’s *t*-test compared with controls. The results represent the mean ± SEM values of three independent experiments. FTB, hydroethanolic extract of *F. thunbergii* Bulbus. FD-4, FITC-conjugated dextran probe. * *p* < 0.05, ** *p* < 0.01, *** *p* < 0.001.

TEER, an established marker of Caco-2 cell barrier function, was used to investigate the effect of FTB on differentiated Caco-2 cells exposed to inflammatory stimulation, as shown in Figure 5 and Supplementary Figure S2. Our findings revealed that FTB did not affect the viability of Caco-2 cells in a concentration range of 6.25–25 μg/mL, with or without IL-6 (Supplementary Figure S2). IL-6 exposure led to a 59% reduction in Caco-2 TEER from the initial value, whereas FTB significantly restored Caco-2 TEER in a concentration-dependent manner starting at a concentration of 12.5 μg/mL (12.5 μg/mL, *p* < 0.01; 25 μg/mL, *p* < 0.001; Figure 5A). To investigate paracellular permeability, we measured the flux of fluorescent molecules across the Caco-2 monolayers. Our results showed that IL-6 increased dextran permeability by 187%, while FTB decreased permeability in a concentration-dependent manner starting at a concentration of 12.5 μg/mL (12.5 μg/mL, *p* < 0.01; 25 μg/mL, *p* < 0.001; Figure 5B). Additionally, FTB restored the levels of TJ proteins, ZO-1, and occludin in Caco-2 cells. The expression level of ZO-1, which decreased by 0.7-fold in the control (*p* < 0.01), was increased to 1.1-fold at 6.25 μg/mL of FTB, 1.4-fold at 12.5 μg/mL of FTB (*p* < 0.05), and 1.5-fold at 25 μg/mL of FTB (*p* < 0.01) (Figure 5C). Moreover, the expression level of occludin, which decreased by 0.7-fold (*p* < 0.01), increased 0.9-fold at an FTB concentration of 6.25 μg/mL, 1.1-fold at 12.5 μg/mL of FTB (*p* < 0.01), and 1.3-fold at 25 µg/mL of FTB (*p* < 0.05) (Figure 5D).

## 4. Discussion

IBD is a condition characterized by recurrent episodes of inflammation in the gastrointestinal tract, caused by an abnormal immune response. Currently, drugs used for the treatment of IBD have limitations due to their side effects, and the duration of treatment. Traditional herbal medicine has been used to prevent and improve various diseases and is attracting attention as an alternative treatment to conventional drugs in IBD research [28]. FTB, a traditional herbal medicine used in Korea, reportedly has several pharmacological effects, including anti-inflammatory effects. However, the effect of FTB on IBD remains unclear. Therefore, we evaluated the pharmacological effects of FTB on DSS-induced ulcerative colitis in mice and its effect on intestinal barrier permeability in vitro.

In this study, we selected the DSS-induced ulcerative colitis mouse model as an animal model to evaluate the effect of FTB on UC. The DSS-induced ulcerative colitis mouse model indicates typical symptoms of ulcerative colitis, including weight loss, severe diarrhea, bloody stool, ulceration of the intestinal mucosa, and shortening of the colon. These symptoms are similar to those of human ulcerative colitis; therefore, they are widely used in colitis research [29]. Although the clinical effective dose (4.8 g/d) of mesalamine for patients corresponds to 800 mg/kg/d for mice [30], the no-observed adverse effect level (NOAEL) of mesalamine in rodents is 200 mg/kg/day in repeated oral dose toxicity studies. In this context, we selected an optimal dose of mesalamine (200 mg/kg) and compared it with a higher concentration (200 mg/kg) of FTB. We investigated the effect of FTB on ulcerative colitis in mice given drinking water supplemented with 4% DSS. Compared with the normal group, the DSS-administered group showed weight loss beginning on day five, whereas the FTB-administered group showed significant weight loss on day eight. Similar to weight loss, DAI was significantly inhibited by the administration of FTB. Additionally, FTB treatment suppressed colon shortening and spleen enlargement. Histological analysis of colonic mucosa showed that FTB protected against damage to the mucosa, submucosa, muscularis, and serosa layers due to DSS treatment. These results indicate that FTB significantly improves DSS-induced ulcerative colitis symptoms.

Oral administration of DSS results in severe damage to the colonic mucosa, increased intestinal permeability, mucosal hypertrophy, inflammatory cell infiltration, and severe intestinal inflammation [31]. Inflammatory cytokines and mediators, such as IL-1b, IL-6, TNF-α, iNOS, and PGE2, are involved in inflammatory responses that induce ulcerative colitis in patients with intestinal inflammation. Furthermore, it plays an important role in the initiation and continuation of colitis [32,33]. Inflammatory factors such as IL-1b, IL-6, TNF-α, iNOS, and COX-2, are frequently expressed in ulcerative colitis models. When these inflammatory factors are excessively secreted, the balance of the body’s immune system is disrupted, leading to intestinal inflammation and ulceration. The results of our study suggest that the increase in inflammatory cell infiltration and inflammatory factors in the N.C. group compared to that in the normal group was reduced in the FTB-treated group. This can be attributed to the protection of intestinal barrier integrity by FTB administration.

The intestinal barrier is a crucial layer that separates the lumen from the external environment, and TJs are considered to be important components of the epithelial boundary [34]. TJs comprise various transmembrane proteins linked to cytoplasmic adapters that enable them to attach to neighboring cells and regulate the passage of water, ions, and other substances. Four integral proteins, namely the claudin family, occludin, junctional adhesion molecule, and tricellulin, cooperate with ZO-1, a cytoplasmic complex protein, to structurally maintain and function TJ [35,36]. Therefore, increased levels of TJ proteins can maintain the structural integrity of the intestinal barrier and, hence, prevent colitis [37]. Consequently, we examined the distribution of occludin and ZO-1 in the colon tissues of mice with DSS-induced ulcerative colitis using IHC, and observed a significant protein decrease in the N.C. group, which was prevented by FTB. It can be inferred that FTB prevents the collapse of TJ in the experimental UC model, thereby reducing the severity of colitis. Moreover, we investigated the effects of FTB on barrier permeability in vitro. At FTB concentrations ranging from 0 to 100 μg/mL, cell viability was observed at all concentrations except for 100 μg/mL, where it decreased. The TEER value is used to evaluate intercellular TJ because it reflects the ability of intestinal mucosal cells to act as a permeation barrier [38]. If TJ is well maintained, the membrane is firmly attached, and the current flowing between cells generates high resistance, resulting in a high TEER value. Conversely, a low TEER value indicates increased permeability. Our study revealed that TEER, which decreased by 59% following IL-6 treatment, increased by 90% in FTB in a concentration-dependent manner. In addition, the FD-4 permeability results, which directly reflect the change in permeability, showed that permeability increased by 188% after IL-6 treatment, but FTB decreased it by 49% in a concentration-dependent manner. These results suggest that the increased resistivity and decreased permeability due to FTB pretreatment can prevent barrier function from collapsing by maintaining the TJ well. Furthermore, FTB increased the expression levels of ZO-1 and occludin, which decreased by 65% and 70%, respectively, to a maximum of 151% and 133%, respectively, due to IL-6. FTB maintains the protein expression levels of ZO-1 and occludin in relation to TJ, suggesting that it may help to prevent TJ collapse and improve intestinal barrier integrity.

ECM remodeling may reflect the integrity of barrier epithelial cell homeostasis [39]. ECM changes in colonic tissue, characterized by increased degradation of ECM components and excessive intestinal fibrosis, are defining features of IBD progression [40]. Unbalanced ECM remodeling disrupts tissue homeostasis, leading to severe tissue destruction and abnormal healing [41]. In this study, we investigated the accumulation of collagen in colon tissue using MT staining and compared the mRNA expression levels of factors related to ECM remodeling. The MT staining images showed increased collagen accumulation in the N.C. group compared to the normal group, which was significantly lowered by FTB. Moreover, the mRNA expression of factors related to ECM remodeling was regulated by FTB. According to a previous study, MMP-7 exacerbates inflammation by damaging the intestinal wall through the cleavage of claudin-7 [42]. MMP-9 participates in the inflammatory response, slows down the epithelial repair process, hinders wound healing, increases endothelial permeability, and activates cytokines and chemokines, including interleukin IL-1β, IL-8, and TGF-β, and neutrophil infiltration into the intestinal tissue [43]. Col3a1 overexpression causes collagen accumulation and intestinal fibrosis [44,45]. Additionally, TIMP-1 is expressed in colitis lesions and is involved in the production of the proinflammatory cytokine IL-10 [46]. Real-time PCR showed that the N.C. group exhibited improved expression of MMPs, Col3a1, and Timp-1 compared to the normal group, which was significantly lowered by FTB. These results suggest that FTB can reduce the symptoms of fibrosis, such as collagen hyperaccumulation, by regulating the mRNA expression of factors related to ECM remodeling.

The mucus layer provides barrier protection by covering the gastrointestinal tract and is one of the first lines of immune defense [47]. TFF3 is a TFF peptide expressed in goblet cells of the small and large intestines [48], and Klf4 plays an important role in the differentiation of goblet cells to regulate intestinal epithelial homeostasis [49,50]. Our results showed that, compared to the reduced number of goblet cells due to DSS-induced ulcerative mucosal collapse in the N.C. group, the number of goblet cells was higher in the FTB-administered groups and did not decrease. The unchanged mRNA expression levels of mucin 2 and Klf3 in all groups and the decreased TFF3 expression level of FTB-administered groups compared to the N.C. group could be related to the prevention of intestinal epithelial TJs of FTB discussed above.

Furthermore, among the nine identified ingredients in this study, several compounds have been reported to possess pharmacological effects. Notably, Michaudel et al. [51] demonstrated that the administration of xanthurenic acid reduced body weight loss, disease activity index (DAI), colon length shortening, and histological score in a DSS-induced experimental colitis mouse model compared to the control group (1). Sipeimine (5) has been shown to protect against lung damage induced by fine particulate matter [52,53], while peimisine (6), peimine (7), and peiminine (8) have exhibited anti-inflammatory effects in LPS-induced RAW264.7 cells [54,55]. Additionally, peimisine (6) and peimine (7) have been reported to have antitussive effects [56,57]. Further investigation is warranted to explore the ingredients that are associated with the factors that influence the UC, such as intestinal barrier integrity, ROS, inflammation, the physical barrier of the intestine, microbiota, and intestinal immunity.

Our results indicate that FTB improves intestinal barrier integrity by preventing damage to the TJs, regulating ECM remodeling, and preventing disability in the mucus layer. Moreover, these results suggest that FTB reduces the infiltration of inflammatory cells in the colon tissue and decreases the mRNA expression of inflammatory factors.

## 5. Conclusions

In conclusion, treatment with FTB suppressed clinical symptoms caused by enteritis, inhibited the infiltration of inflammatory cells into the intestinal wall, reduced the expression of inflammation-related mRNA and ECM remodeling, prevented damage to the intestinal mucosa, inhibited TJ disruption, reduced barrier permeability, and alleviated UC induced by DSS. These findings suggest that FTB can be used as an effective agent for barrier protection in patients with UC.

## Figures and Tables

**Table 1:** Identified phytochemicals in FTB by UPLC-MS/MS.

No.	Theoretical *m*/*z*	Measured *m*/*z*	Error (ppm)	Adduct	R_t_ (min)	Formula	Fragments (*m*/*z*)	Identifications
1	206.0448	206.0447	−0.496	[M+H]^+^	4.78	C_10_H_7_NO_4_	206, 178	Xanthurenic Acid *
2	444.3108	444.3102	−1.405	[M+H]^+^	6.48	C_27_H_41_NO_4_	426, 114	Yibeissine [21]
3	594.4000	594.3992	−1.370	[M+H]^+^	7.08	C_33_H_55_NO_8_	576	Peiminoside [21]
4	592.3844	592.3838	−0.834	[M+H]^+^	7.17	C_33_H_53_NO_8_	574	Sipeimine glucoside [22]
5	430.3316	430.3312	−0.914	[M+H]^+^	7.27	C_27_H_43_NO_3_	430	Sipeimine *
6	428.3159	428.3155	−0.931	[M+H]^+^	7.5	C_27_H_41_NO_3_	428	Peimisine *
7	432.3472	432.3466	−1.320	[M+H]^+^	7.77	C_27_H_45_NO_3_	432, 414	Peimine *
8	430.3316	430.3310	−1.410	[M+H]^+^	8.19	C_27_H_43_NO_3_	430, 412	Peiminine *
9	398.3417	398.3412	−1.470	[M+H]^+^	12.15	C_27_H_43_NO	398	Solanidine *

* Compared with the retention time (Rt) and mass spectra of genuine standards.

## Data Availability

Not applicable.

## Supplementary Materials

The following supporting information can be downloaded at https://www.mdpi.com/article/10.3390/nu15122810/s1, Figure S1: UPLC-MS/MS analysis of FTB; Figure S2: Effects of IL-6 and FTB on cell viability of Caco2 cells; Figure S3: Original Western blot for three repeats; Table S1: Taqman probe information of target genes used in real-time PCR. Reference [58] is cited in the supplementary materials.
