# Supplementary material for: Hydroethanolic Extract of Fritillariae thunbergii Bulbus Alleviates Dextran Sulfate Sodium-Induced Ulcerative Colitis by Enhancing Intestinal Barrier Integrity
Source: Nutrients. 2023 Jun 20;15(12):2810. doi: 10.3390/nu15122810 (PMC10302580; doi:10.3390/nu15122810)
Supplement: Supplementary file 1 [file nutrients-15-02810-s001.zip › nutrients-2385866-supplementary.pdf]

Supplementary Material and Methods

Identification of phytochemicals by Ultra-high performance liquid chromatography-tandem mass spectroscopy (UPLC-MS/MS)

The Q-Exactive mass spectrometer was operated in positive and negative ionization switching modes using a heated electrospray ionization (HESI) source, as described previously [1]. The Freestyle v.1.3. and TraceFinder v.3.2 softwares were used for data acquisition and analysis (Thermo Fisher Scientific, Waltham, MA, USA).

Colitis severity evaluation

The disease activity index (DAI) was assessed daily after DSS treatment as the sum of scores for the percentage of weight loss (none, 0; 1–5%, 1; 5–10%, 2; 10–15%, 3; more than 15%, 4), stool consistency (normal, 0; loose stools, 2; watery diarrhea, 4), and rectal bleeding (no bleeding, 0; slight bleeding, 2; gross bleeding, 4).

Real-time PCR

Total RNA and cDNA were prepared from the colon tissues using the RNeasy kit (Qiagen, Hilden, Germany) and the High-Capacity cDNA Reverse Transcription Kit (Thermo Fisher Scientific), respectively, according to the manufacturer's instructions. Real-time PCR was performed using TaqMan Universal PCR Master Mix (Applied Biosystems, Foster City, CA, USA) and TaqMan primer probes (Thermo Fisher Scientific) for target genes in an ABI 7500 Real-Time PCR system (Applied Biosystems). The information for each TaqMan probe used in the real-time PCR is listed in Supplementary Table S1. The relative mRNA expression

of target genes was normalized using the ΔΔCt method and presented as fold-change. Results were obtained from three independent experiments.

Western blot analysis

Caco-2 cells were cultured in a 6-well plate at a seeding density of 3×10^5 cells/well. The medium was refreshed every two days until complete differentiation was achieved after 21 days. After differentiation, cells were pretreated with various doses of FTB for 1 h and then exposed to 50 ng/mL IL-6 for an additional 24 h. Proteins were extracted using RIPA lysis buffer. The proteins were quantified using a BCA kit, and equal amounts were separated on 6% and 12% SDS-PAGE gels and transferred to nitrocellulose membranes. The membranes were blocked and incubated overnight at 4°C with a 1:1000 dilution of ZO-1(Cell Signalling, 5406S, Danvers, MA, USA) or occludin (Cell Signalling, 91131S) antibodies. The membranes were then incubated with secondary antibodies and visualized using a ChemiDoc Touch Imaging System (Bio-Rad, Hercules, CA, USA). The band density was normalized to that of β-actin. Results were obtained from three independent experiments.

Reference

[1] A. Lee, H. Yang, T. Kim, H. Ha, Y.-H. Hwang, Identification and pharmacokinetics of bioavailable anti-resorptive phytochemicals after oral administration of Psoralea corylifolia L, Biomedicine & Pharmacotherapy 144 (2021) 112300.

Table S1. Taqman probe information of target genes used in real-time PCR

No.	Gene symbol	Assay ID of Taqman probe	NCBI Reference Sequence
1	Il1b (IL-1β)	Mm00434228_m1	NM_008361.3
2	IL-6	Mm99999064_m1	NM_031168.1
3	Tnf (TNF-α)	Mm00443258_m1	NM_001278601.1
4	Ptgs2 (Cox-2)	Mm00478374_m1	NM_011198.3
5	Nos2 (iNOS)	Mm00440502_m1	NM_010927.3
6	Mmp7 (MMP-7)	Mm00487724_m1	NM_010810.4
7	Mmp9 (MMP-9)	Mm00442991_m1	NM_013599.3
8	Col3a1	Mm00802300_m1	NM_009930.2
9	Timp1	Mm01341361_m1	NM_001044384.1
10	Muc2	Mm01276696_m1	NM_023566.3
11	Tff3	Mm00495590_m1	NM_011575.2
12	Klf4	Mm00516104_m1	NM_010637.3
13	Actb (β-actin)	Mm00607939_s1	AK078935.1

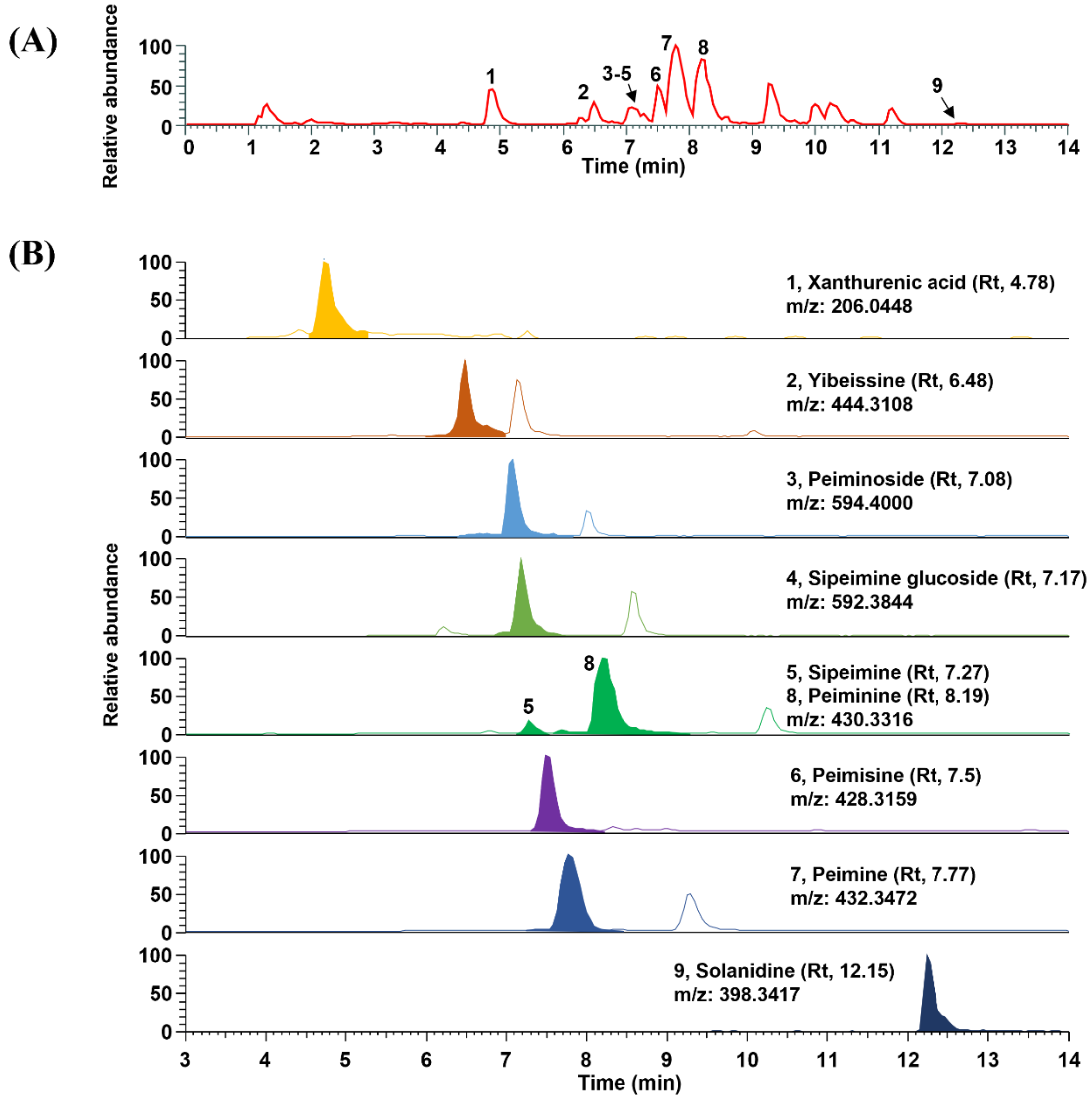

Figure S1. UPLC-MS/MS analysis of FTB. (A) Base peak chromatogram of FTB with positive ionization mode on UPLC-MS/MS; (B) Extracted ion chromatogram of identified phytochemicals with retention time (R_t) between 3 and 14 min.

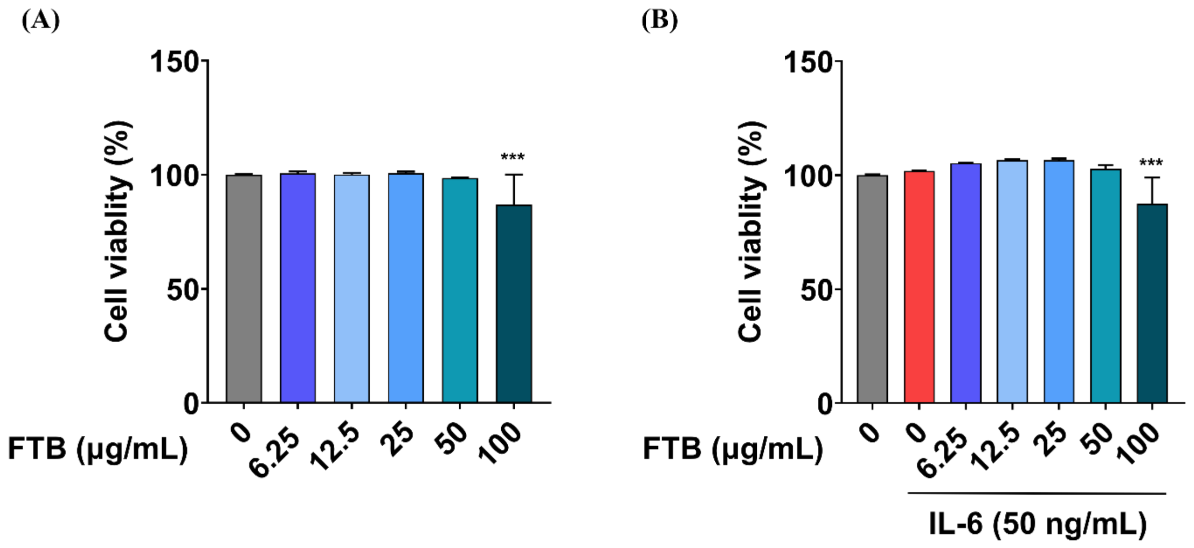

Figure S2. Effects of IL-6 and FTB on cell viability of Caco2 cells. (A) Cell viability of FTB; (B) Cell viability of FTB with Interleukin (IL)-6. Cell viability is represented as the percentage relative absorbance compared with controls. All data were analyzed using one-way ANOVA with Dunnett's post-hoc test compared to controls. The results represent the mean ± SEM values of three independent experiments. FTB, hydroethanolic extract of *F. thunbergii* Bulbus. *** $p < 0.001$.

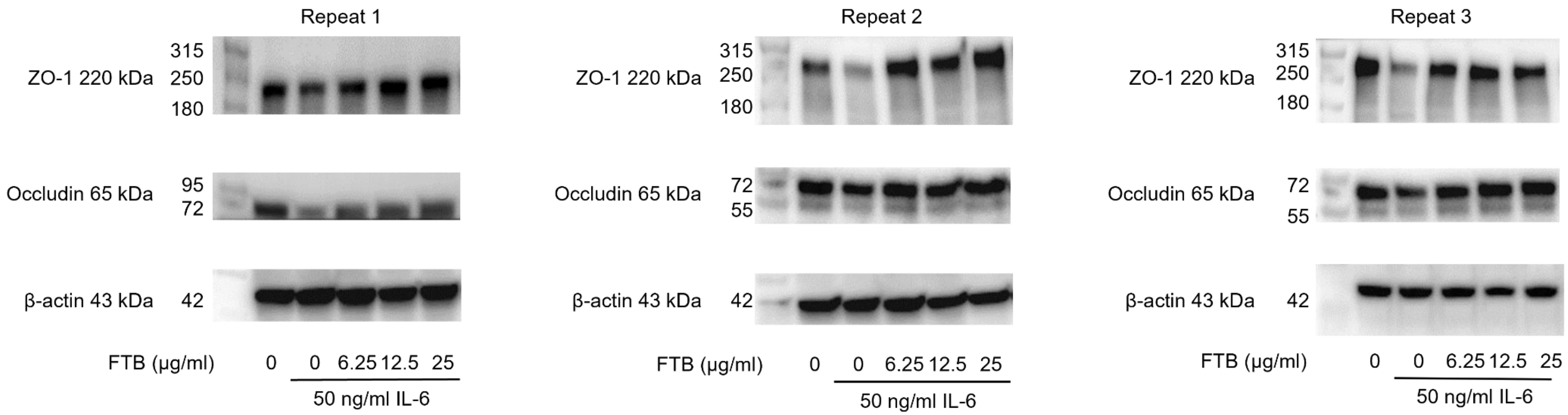

Figure S3. Original western blot for three repeats
